# Role of atrial natriuretic peptide (ANP) in the regulation of insulin secretion and vitality of pancreatic ß cells

**DOI:** 10.1186/2050-6511-16-S1-A92

**Published:** 2015-09-02

**Authors:** Sabine Tauscher, Hitoshi Nakagawa, Katharina Völker, Birgit Gaßner, Saskia Pröhl, Michaela Kuhn

**Affiliations:** 1 Institute of Physiology, University of Würzburg, Würzburg, Germany; 2 First Department of Internal Medicine, Nara Medical University, Kashihara, Japan

## Background

The cardiac hormone atrial natriuretic peptide (ANP) increases intracellular cyclic GMP levels via activation of the guanylyl cyclase-A (GC-A) receptor and exerts important endocrine effects regulating arterial blood pressure and intravascular volume. Besides these well-known effects, several studies suggest a role for ANP and its receptor in the endocrine pancreas. For instance, ANP infusion enhanced insulin plasma levels in healthy subjects [1]. Even more low plasma ANP levels predict the development of diabetes type II [2]. Also it has been shown that ANP can stimulate the function and the growth of cultured pancreatic ß cells [3,4]. Since these studies were conducted with synthetic ANP, the question remains whether the endogenous ANP/GC-A system modulates insulin secretion or the growth and vitality of pancreatic ß cells under (patho)physiological conditions. Therefore, here we generated and studied a novel mouse model with conditional, cell-restricted deletion of the GC-A receptor in cells (GC-A^fl/fl^;Rip-Cre^+/-^: ß GC-A KO mice). The Rip-Cre mouse line was provided by Dr. Pedro Herrera [5]. The GC-A^fl/fl^ mice were previously generated in our group [6].

## Methods and results

Our first aim was to characterize whether ANP modulates ß cell vitality and function under pathological conditions of insulin resistance. Five weeks old ß GC-A KO and control littermates were fed with a high-fat (60% energy from fat, HFD) versus a normal diet (10% energy from fat, ND) during 3 months. The HFD induced a genotype-independent increase in body weight, arterial blood pressure and fasted blood glucose levels. Moreover, all mice showed a pathological oral glucose tolerance test (oGTT), confirming an insulin resistant state. In control mice, immunostainings of insulin and morphometrical analyses showed an increase in the islet area, the total area of ß cells per islet and in the number of ß cells per islet after HFD. Since the area of single ß cells remained the same under ND and HFD, these results demonstrate that the observed increase in ß cell mass was caused by ß cell proliferation and not by hypertrophy. Strikingly, ß cell proliferation in response to HFD-provoked insulin resistance was abolished in ß GC-A KO mice.

As mentioned above, low plasma levels of ANP predict development of future diabetes and glucose progression in the elderly, suggesting a (co)causal role of deficient ANP/GC-A signalling in diabetes development [2]. To study whether chronic ß cell ANP/GC-A dysfunction alters insulin-dependent glucose homeostasis, we analyzed female ß GC-A KO and control mice at 10-15 months of age. Fasted insulin plasma levels were not different between genotypes. Surprisingly, the oGTT showed a better glucose handling in the ß GC-A KO mice, with lower plasma glucose levels at all time-points and a significantly lower area under the curve (AUC). Insulin sensitivity was not different between genotypes. However, glucose-stimulated insulin release was enhanced in the ß GC-A KO mice.

## Conclusions

Our data indicate that ANP/GC-A signalling contributes to enhanced β-cell proliferation in situations of insulin resistance, for instance under HFD. The molecular mechanism of the improved glucose-dependent insulin secretion in the older ß GC-A KO mice is being addressed in our current investigations. Our study contributes to the recent appreciation of the metabolic roles of ANP and suggests an endocrine axis between the heart and the endocrine pancreas.
